# Supplementary material for: CD63-Mediated SARS-CoV-2 RBD Fusion Neoantigen DNA Vaccine Enhances Antitumor Immune Response in a Mouse Panc02 Model via EV-Targeted Delivery
Source: Vaccines (Basel). 2025 Sep 16;13(9):977. doi: 10.3390/vaccines13090977 (PMC12474169; doi:10.3390/vaccines13090977)
Supplement: Supplementary file 1 [file vaccines-13-00977-s001.zip › Supplemental Figures.pdf]

Supplemental Figures

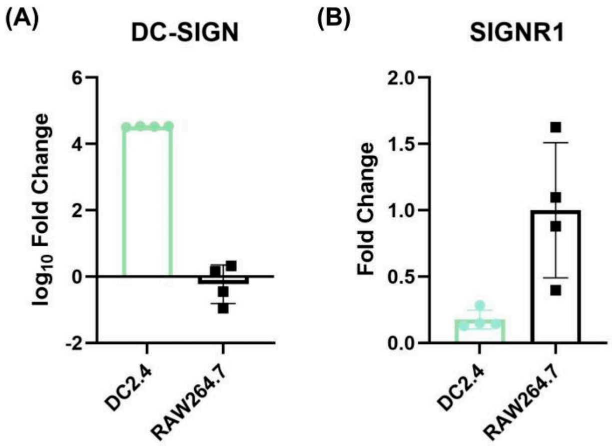

Figure S1. Expression levels of DC-SIGN(A) and SIGNR1(B) in DC2.4 and RAW264.7 cells. Quantitative real-time PCR was used to compare the mRNA expression of DC-SIGN (CD209a) and SIGNR1 (CD209b) between murine dendritic cell line DC2.4 and macrophage-like cell line RAW264.7. Data are presented as fold change relative to the housekeeping gene, normalized to RAW264.7 as control.

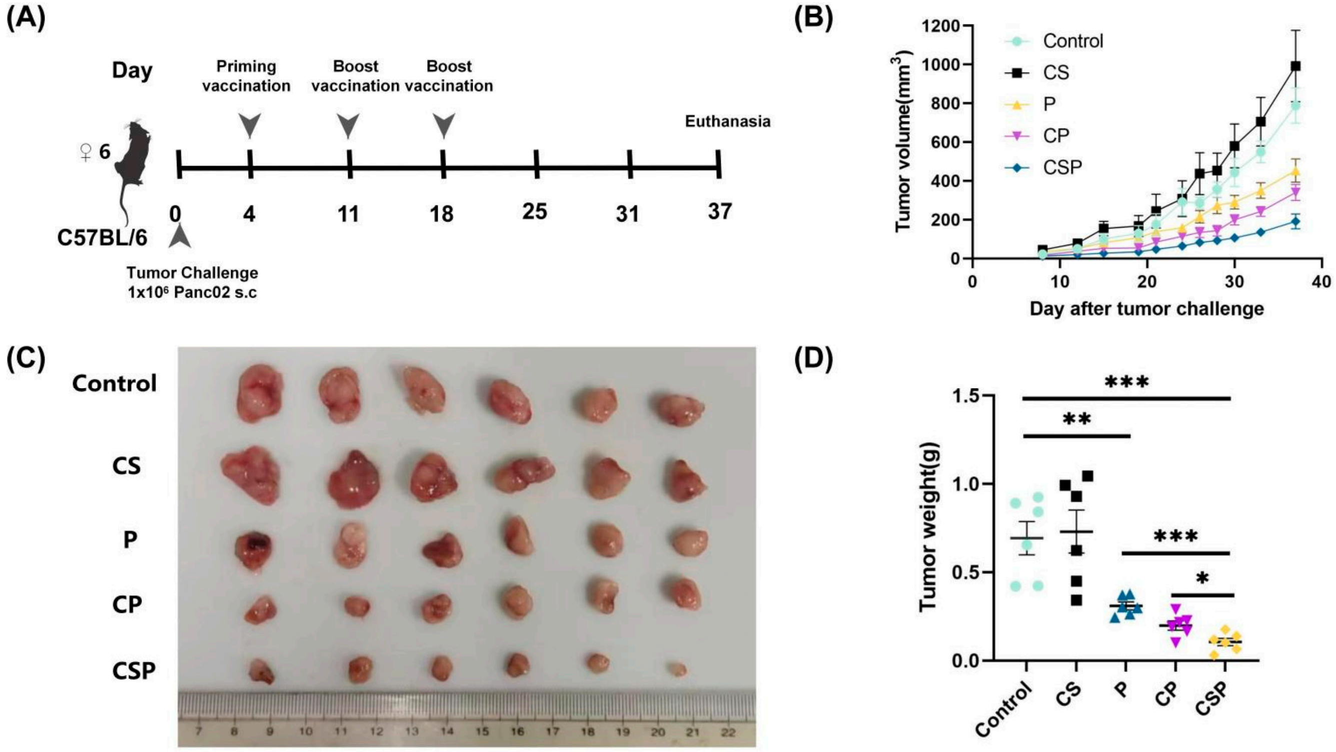

Figure S2. pCSP Inhibits Tumor Growth in a Subcutaneous Mouse Tumor Model. (A) Experimental timeline for the subcutaneous mouse tumor model. (B) Tumor growth curves for different groups . (C) Images of subcutaneous tumors excised from each group. (D) Tumor weight analysis showing significant reductions in tumor weight in the CSP group compared to other groups.*P < 0.05, **P < 0.01, and ***P < 0.001,n=6.

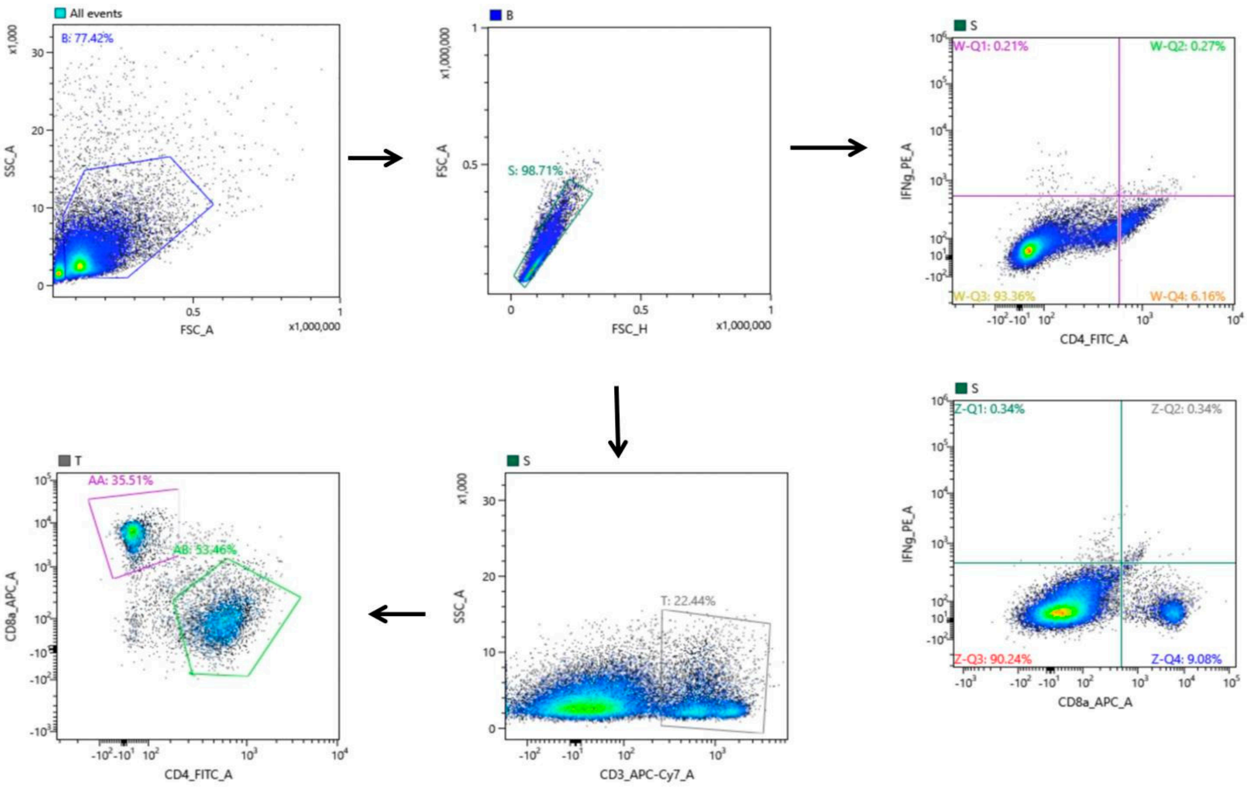

Figure S3. Flow-cytometry gating strategy (ICS) on splenocytes from immunized mice. Splenocytes were harvested after immunization and analyzed by intracellular cytokine staining. Gating proceeded as indicated by arrows: (1) FSC-A vs SSC-A to exclude debris and define lymphocytes; (2) FSC-A vs FSC-H to select singlets; (3) $CD3^+$ T cells identified (CD3 APC-Cy7 vs SSC-A); (4) subdivision into $CD4^+$ and $CD8^+$ subsets; (5) IFN-γ^+ events quantified within $CD4^+$ or $CD8^+$ gates with thresholds set by FMO controls. Representative gates and event frequencies are shown.

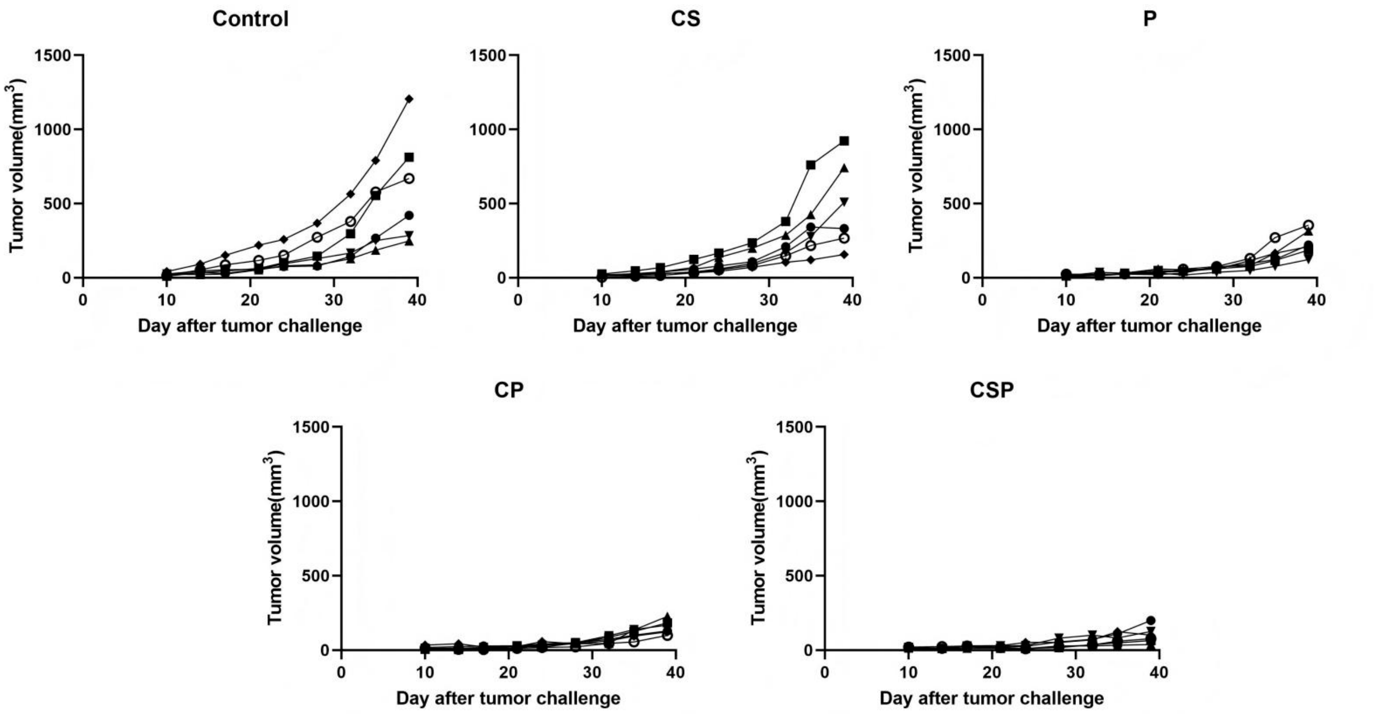

Figure S4 related to Figure 5C. Individual spider plots of tumor growth. Longitudinal tumor volumes (mm^3) for each mouse are shown for the control, CS, P, CP, and CSP cohorts as days after tumor challenge. Each line represents one mouse; symbols mark successive measurement days. Tumor measurements were performed under blinded conditions and followed until the predefined study endpoint.

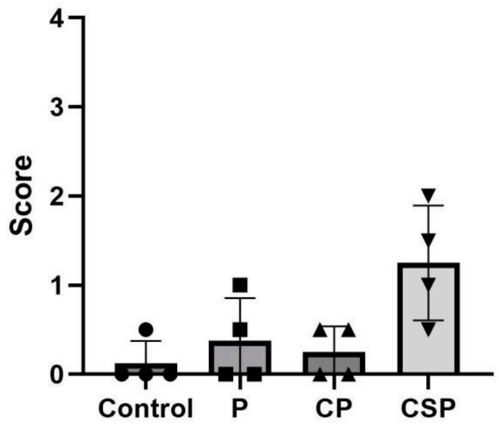

Figure S5. Semi-quantitative lung inflammation scores. H&E-stained lung sections from Control, P, CP, and CSP groups were scored under blinded conditions on a 0–4 scale per high-power field (HPF) for alveolar neutrophils, interstitial infiltration, peribronchial/perivascular cuffing, intra-alveolar exudate, hemorrhage, and septal thickening. Bars = mean ± SD; symbols = individual mice. The results showed that compared with other groups, the CSP group mice had significantly more mild inflammation in the lungs.
